# Supplementary material for: CHOLINERGIC MODULATION OF CELLULAR RESONANCE IN NON-HUMAN PRIMATE HIPPOCAMPUS
Source: bioRxiv. 2025 Jan 11:2025.01.10.632495. Preprint. [Version 1] doi: 10.1101/2025.01.10.632495 (PMC11741476; doi:10.1101/2025.01.10.632495)
Supplement: Supplement 1 [file NIHPP2025.01.10.632495v1-supplement-1.pdf]

bioRxiv preprint doi: https://doi.org/10.1101/2025.01.10.632495; this version posted January 11, 2025. The copyright holder for this preprint (which was not certified by peer review) is the author/funder, who has granted bioRxiv a license to display the preprint in perpetuity. It is made available under aCC-BY 4.0 International license.

Supplemental Figure 1.

A. Violin plot showing the Preferred Resonance Frequency of all neurons recorded.

B. Histogram of the Preferred Resonance Frequency of all neurons recorded as in A. Inset, QQ plot comparing the distribution of the dataset in A to a theoretical normal distribution.

C. Preferred Resonance Frequency of CA1 neurons recorded at different temperatures as indicated. Two tailed unpaired t test, $p=0.0025$

D. Preferred Resonance Frequency of CA1 neurons in current clamp at different baseline voltages, as indicated. A one-way ANOVA revealed a significant difference ($F = 23.81$, $p < 0.0001$), followed by Tukey's multiple comparison test.

Supplemental Figure 2.

A. Violin plot displaying the Resting Membrane Potential of non-resonator and resonator cells. A two-tailed Mann-Whitney test revealed no significant difference between the groups ($p = 0.5958$).

B. Violin plot displaying the membrane resistance of non-resonator and resonator cells. A two-tailed Mann-Whitney test revealed no significant difference between the groups ($p = 0.2816$).

C. Scatter plot showing the relationship between membrane resistance and maximal impedance in non-resonator and resonator cells.

D. Scatter plot showing the relationship between membrane resistance and Preferred Resonance Frequency in non-resonator and resonator cells.

E. Scatter plot showing the relationship between maximal impedance and Preferred Resonance Frequency in non-resonator and resonator cells.

Supplemental Figure 3.

A. Rescaled values of impedance ([0 max]) from non-resonator cells before and after treatment with carbachol and binned around integer frequencies and compared using a 2-way ANOVA (Frequency x before/after carbachol: $F(1.718, 20.62) = 23.85$, $p<0.001$), followed by Tukey's multiple comparisons test.

bioRxiv preprint doi: https://doi.org/10.1101/2025.01.10.632495; this version posted January 11, 2025. The copyright holder for this preprint (which was not certified by peer review) is the author/funder, who has granted bioRxiv a license to display the preprint in perpetuity. It is made available under aCC-BY 4.0 International license.

B. Rescaled values of impedance ([0 max]) from resonator cells before and after treatment with carbachol and binned around integer frequencies and compared using a 2-way ANOVA (Frequency x before/after carbachol: $F(1.328, 11.95) = 1.353$, $p=0.28$), followed by Tukey's multiple comparisons test.

C. Histogram of the Preferred Resonance Frequency of all neurons treated with carbachol.

D. QQ plot comparing the distribution of the dataset in C to a theoretical normal distribution

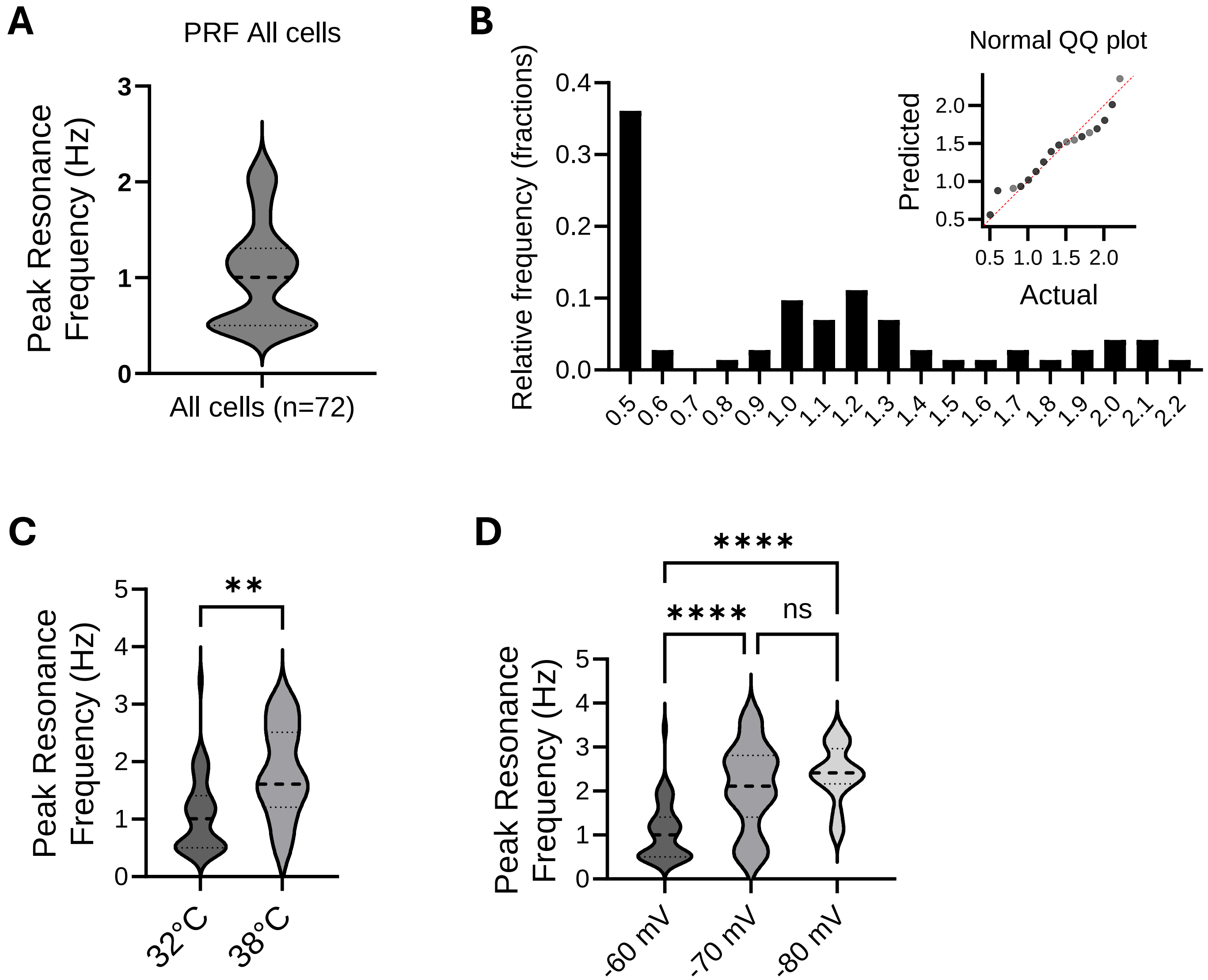

Supplemental Figure 1

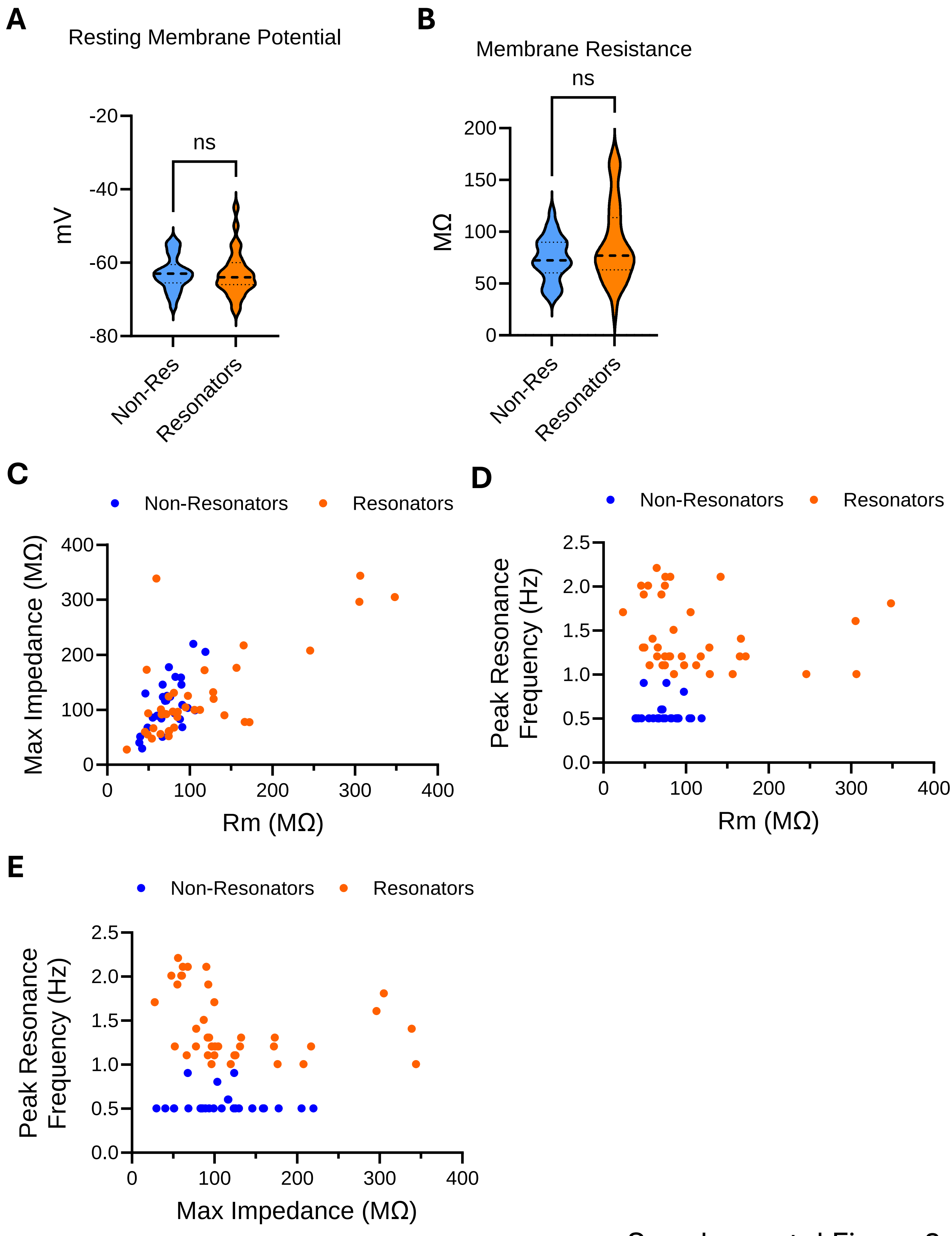

Supplemental Figure 2

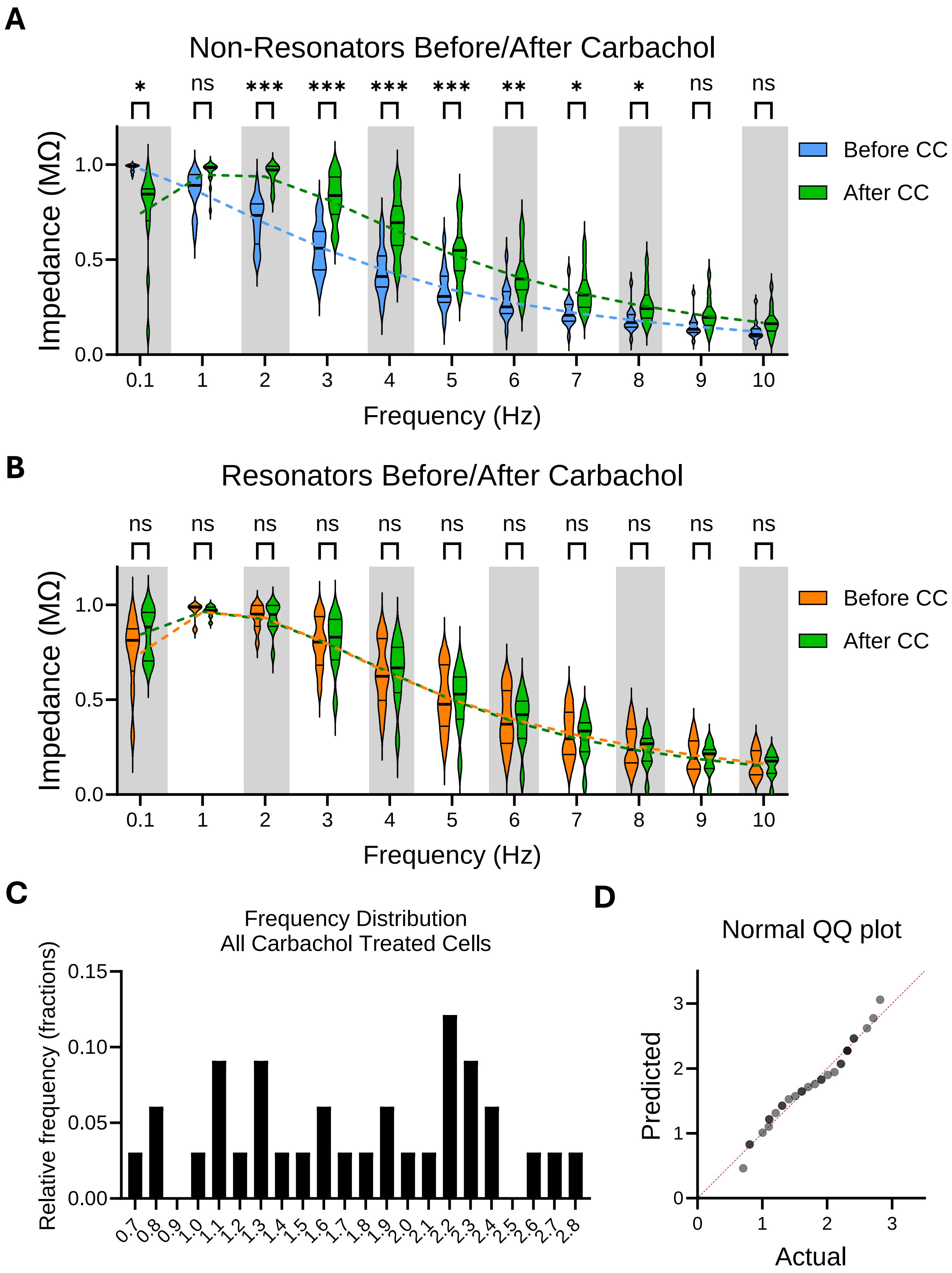

Supplemental Figure 3
